# The Life Satisfaction Index-A (LSI-A): Normative Data for a General Swedish Population Aged 60 to 93 Years

**DOI:** 10.2147/CIA.S275387

**Published:** 2020-11-02

**Authors:** Sölve Elmståhl, Johan Sanmartin Berglund, Cecilia Fagerström, Henrik Ekström

**Affiliations:** 1 Division of Geriatric Medicine, Department of Clinical Sciences in Malmö, Lund University, Lund, Sweden; 2 Blekinge Institute of Technology, Department of Health, Karlskrona, Sweden; 3 Department of Health and Caring Science, Linnaeus University, Kalmar, Sweden

**Keywords:** life satisfaction, LSI-A, well-being normative data, population study, elder

## Abstract

**Purpose of Study:**

To gain Swedish norm value for the Life Satisfaction Index-A (LSI-A) in a population 60–93+ years old stratified for sex and age and to relate these norm values with respect to number of chronic diseases and functional impairment.

**Materials and Methods:**

The study population included a random sample of 2656 men (45.7%) and 3159 (54.3%) women from the longitudinal national studies’ “Good Aging in Skåne” (GÅS) and SNAC-B, both part of the Swedish National Study on Aging and Care (SNAC). Data on Neugartens Life Satisfaction Index-A (LSI-A), medical history, activities of daily life (ADL) and socio-demographics were collected through structured interviews and questionnaires.

**Results:**

Men scored significantly higher than women; 28.5, sd=6.9, and 27.3, sd=6.6, respectively, out of maximum 40 points. For both genders the scores decreased with age, mean score 6.0 points, lower for men and 7.1 points lower for women between 60 and 93+ years. The highest score was noted for healthy individuals where both men and women scored 29.5 points, sd=6.2. Increased number of chronic diseases and dependency in ADLs were associated with lower LS.

**Conclusion:**

Norm values here presented may facilitate assessments and evaluation of life satisfaction in the general elder population and as reference values to clinical trials. Female sex, rising age, morbidity and impaired functional ability were all associated with impaired LS.

## Introduction

Life satisfaction (LS) in the concept of subjective well-being (SWB), is an individual’s cognitive and emotional evaluation at a particular point in time which reflects to what extent social and psychological needs have been met.^1^,^2^ As a theoretical construct life satisfaction cannot be measured directly but assessed as a latent variable, most often through interviews or questionnaire. The Neugartens Life Satisfaction Index-A (LSI-A), one of the most frequently used instruments measuring LS, was constructed in 1961 during a research program aiming to be a valid and reliable instrument not restricted in time. The intention was to measure life satisfaction referring to the individual’s own references relatively independent from other social and psychological measures such as social networks or levels of activity.^3^

In the fields of geriatrics and gerontology the concept of LS is not merely the absence of disease or disability but comprises social and psychological needs and the assessment LS has become important in evaluating successful ageing.^4^,^5^ Bearing in mind the global demographic situation with an ever increasing proportion over 65, it is easy to understand that LS among older people has increasingly caught the attention of social scientists and policy makers.^6^ So far, normative values for LSI-A in the elder general population stratified for age and gender is lacking.

Normative data could be useful from a public health perspective on a national level in the comparison and interpretation of individual or group scores on LSI-A. They can be a guide assessing whether an individual’s score or groups' mean score is above or below average in relation to age, gender, or as here presented, on the basis of number of chronical diseases or functional impairment (dependency in ADL), conditions which have been shown to be among the most significant predictors for a decline in LS.^7–11^ At an international level, norm values can be the basis for comparing variations between countries or larger regions and underpin further studies on how medical, psychological, socio-cultural differences and living conditions may influence LS.^12–15^ Such a comparison could also be a help for policy makers evaluating social welfare projects or as a guide for better targeted interventions with the aim of improving living conditions for the elderly.^6^ Normative data could, from a medical perspective, be used as an outcome variable in clinical case control studies and trials.

Medical, psychological and social predictors for LS among elderly have been reported in numerous studies.^10^,^16–19^ Despite this, norm values for LSI-A in a general elderly population has not been published before.

Thus, the overarching aim of this paper was firstly to present norm values for healthy individuals in an elder general population stratified by age and sex. Secondly, to present norm values for elder individuals affected by one or more chronical diseases or functional impairment, stratified by age and sex.

## Materials and Methods

### Study Population and Research Context

In this cross-sectional study baseline data was retrieved from the ongoing longitudinal projects Good Aging in Skåne (GÅS), and SNAC-B, part of the Swedish National Study on Aging and Care (SNAC).^20^,^21^ The study comprises both urban and rural areas from the southern part of Sweden.

In 2001 participants in nine age cohorts: 60, 66, 72, 81, 84, 87, 90 and 93 years old were randomly drawn from six municipalities using the national municipality population registers. In the GÅS study, two additional randomizations from the 60 and 81 years cohorts were done in 2007 and 2013. In all 11,377 individuals were invited by letter and 7190 (63%) agreed to participate in the projects. The only exclusion criteria was not understanding Swedish. Out of these 7190 individuals, 1063 participants were excluded; 644 did not complete the medical examination, 150 were diagnosed with dementia and another 269 scored less than 18 points on the MMSE test,^22^ indicating a severe cognitive impairment or dementia.^23^ Among the remaining 6127 individuals 467 had at least one unanswered LSI-A question and in order to reduce this internal attrition, participants with up to 5 (25%) missing questions were subjected to mean imputation on an individual level, where means of non-missing values for the specific item was used to impute a total score. This imputation included 155 (2.7%) individuals while those 312 individuals with more than 5 unanswered questions were excluded. Out of the 7190 individuals who agreed to participate, the final study sample then consisted of 5815 (80.1%) participants, 2656 (45.7%) men and 3159 (54.3%) women (Table 1). In total, the external and the internal loss after imputation amounted to 5562 subjects 48.9%.

**Table 1:** Life Satisfaction Index (LSI-A) norm values for a general Swedish population in nine age cohorts between 60–93 Years, N=5815

	n (%)	mn	SD	CI 95%	q1	md	q3	Min–Max	At Ceiling n (%)	Missing n (%)
All	5815	27.8	6.9	27.6–28.0	24.0	29.0	33.0	3–40	49 (0.8)	
Men	2656 (45.7)	28.5	6.6	28.2–28.7	25.0	30.0	33.0	3–40	23 (0.8)	
Women	3159 (54.3)	27.3	7.1	27.0–27.5	23.0	28.0	33.0	3–40	26 (0.8)	
Age, (years)										
60	2481 (42.7)	29.2	6.8	28.9–29.4	25.1	30.0	34.0	3–40	31 (1.2)	
66	908 (15.6)	28.7	6.1	28.3–29.1	25.0	30.0	33.0	4–40	5 (0.5)	
72	414 (7.1)	27.6	6.8	27.0–28.3	24.0	29.0	32.0	3–40	2 (0.5)	
78	346 (6.0)	26.5	6.4	25.9–27.2	22.0	27.0	31.0	6–40	1 (0.1)	
81	879 (15.1)	26.7	6.7	26.2–27.1	22.0	28.0	32.0	6–40	5 (0.5)	
84	335(5.8)	25.0	6.9	24.3–25.8	20.0	25.0	30.0	6–40	2 (0.6)	
87	219 (3.8)	23.8	7.3	22.8–24.8	18.9	24.0	29.0	3–40	2 (0.8)	
90	161 (2.8)	24.2	7.0	23.1–25.3	19.5	24.9	29.2	3–40	1 (0.5)	
93+	72 (1.2)	23.0	7.2	21.3–24.7	18.0	23.0	28.2	6–37	-	
Marital status										19 (0.3)
Living alone	2146 (36.9)	25.5	7.1	25.2–25.8	21.0	26.0	31.0	3–40	11 (0.5)	
Cohabiting	3650 (62.8)	29.2	6.4	29.0–29.4	26.0	30.0	34.0	3–40	38 (1.0)	
Education										40 (0.7)
Elementary school	2722 (46.8)	26.7	6.9	26.4–27.0	22.0	28.0	32.0	3–40	16 (0.6)	
Secondary school	1696 (29.2)	28.2	6.9	27.9–28.5	24.0	29.5	33.0	3–40	16 (0.9)	
University	1357 (23.3)	29.6	6.6	29.2–29.9	26.0	31.0	35.0	5–40	16 (1.2)	
ADL										98 (1.7)
Independent	3901 (67.1)	29.0	6.4	28.8–29.2	25.0	30.0	30.0	3–40	43 (1.1)	
Dependent in IADL	706 (12.1)	25.8	7.2	25.3–26.4	21.0	27.0	32.0	5–40	2 (0.3)	
Dependent in PADL	1110 (19.1)	24.8	7.3	24.4–25.3	20.0	26.0	30.0	3–40	3 (0.3)	
Number of chronic diseases										
None	1950 (33.5)	29.5	6.2	29.2–29.8	26.0	31.0	34.0	3–40	24 (1.2)	
One	2015 (34.7)	28.1	6.6	27.9–28.4	24.0	29.0	33.0	3–40	15 (0.7)	
Two	1174 (20.2)	26.3	7.2	25.6–26.7	21.0	27.0	32.0	6–40	7 (0.6)	
Three or more	676 (11.6)	24.5	7.5	24.0–25.1	19.0	26.0	30.0	3–40	(0.4)	

Structured interviews including questions about past and present health, medication and cognitive tests as well as medical and psychological examinations were carried out by trained medical staff according to predefined research protocols. Self-reported questionnaires were used to obtain data on socio-demographics, activities of daily living (ADL) and life satisfaction (LS). Assessments took place either at the research outpatient clinics or in participants’ home. The latter to minimize any selection bias and to accommodate participants who, due to health reasons, had difficulties getting to the research centers. In this study we added two socio-demographic variables; marital status and education in addition to number of chronical diseases and ADL, variables that in previous studies have shown to be of importance in assessing LS.^24–26^

### Demographics

Descriptive socio-demographic variables included age, sex, marital status, level of education. Marital status was dichotomized into cohabiting (married/cohabitant) or living alone (unmarried/divorced/widowed). Level of education was categorized into elementary school or less, secondary school, or university studies.

### Self-Rated Life Satisfaction (LS)

LS was assessed using Neugartens’ Quality of Life scale (LSI-A).^3^ LSI-A is multidimensional and consists of 20 attitude questions reflecting perceived LS in old age, for example “As I grow older, things seem better than I thought they would be” and “ I have made plans for things I´ll be doing a month or a year from now” (see Appendix A). From a three-point Lickert scale each question could be answered with “disagree”, “do not know” or “agree”, scored 0, 1.0 and 2.0 respectively. Twelve questions were positively formulated and the “agree” answers were scored 2.0, while 8 questions negatively formulated were scored 0 on the “agree” answer. The total score ranged between 0 and 40 and a higher score indicated a better LS.

LSI-A covers 5 domains which are believed to be essential for a cognitive, judgmental, global evaluation of life satisfaction; the dimensions zest and mode tune reflect how life satisfaction is perceived at the present time while the dimensions resolution and fortitude, positive self-concept, congruence between desired and achieved goals in life cover both the past and thoughts about the future and life in general. That is, a person who judges his life satisfaction as high according to the LSI-A has a positive self-image and an optimistic attitude towards life and believes that the goals he set in life have been reached, mostly finds himself in an optimistic or good mood and expecting something good of the future. Since its introduction, LSI-A has been frequently used in aging research although a number of methodological analyses have failed to recreate the five-factor solution suggested by Neugarten et al,^27^,^28^ which is probably the main reason why LSI-A mostly is used as a one-dimensional instrument. Validity and reliability for internal consistency were established by Neugarten et al,^3^ results that have been confirmed in a meta-analysis by Wallace & Wheeler^29^ including 34 journal articles showing an average reliability of α = 0.79 (sd= 0.1, md=0.79), and by Lobello, Underhill & Fine^30^ presenting Cronbach’s α between 0.85 and 0.92. In the present study high internal consistency was indicated for the whole study population; n=5815, Cronbach’s α = 0.8.

### Activities of Daily Living (ADL)

The ADL Staircase was used to assess functional capacity. The instrument, which is an extended version of the ADL index developed by Katz,^31^ includes five items of personal activities of daily living (P-ADL); feeding, transferring, toileting, dressing, bathing and four instrumental ADL items (I-ADL); cooking, transportation, shopping, cleaning. The assessment was recorded on a three-point scale (independent, partly dependent and dependent), with dependence defined in terms of assistance from another person. After dichotomization based on the principles stated in the original instrument manual,^32^ participants were for each activity categorized as functionally independent or dependent. Finally, dependency in IADL included participants dependent in at least one IADL but independent in all PADLs, and dependency in PADL those participants who were dependent in at least one PADL dependent or not in IADL. The ADL staircase has shown to be a reliable and valid instrument for the assessment of older people’s daily functional ability.^33^

### Morbidity

Morbidity considered illness according to the ICD-10 classification in one or more of the categories of cancer, fracture, heart, lung, neurological, endocrinological or psychiatric diseases; where cancer included all sorts of malign tumors; fractures included hip, vertebrae and wrist fracture; heart disease included myocardial infarction, heart failure angina pectoris, arrhythmia and PTCA (Percutaneous transluminal coronary angioplasty) or other heart surgeries; and lung disease included COPD, asthma or tuberculosis. Neurological disease included stroke (cerebral hemorraghia, infarction), RIND, TIA, Parkinson’s disease or epilepsy; endocrinological diseases included diabetes, hypo- or hyper-thyroidism and psychiatric diseases included depression.

### Ethics

The study was conducted in accordance with the Helsinki Declaration and approved by the regional ethics committee at Lund University 2010–2012, registration no. LU 744–00. All participants provided a written consent to participate and to allow retrieval of information from the National Patient Register medical records.

### Data Analyses

Normative data are provide in the form of means, standard deviations, confidence intervals, quartiles, minimum and maximum scores and proportions at ceiling (maximum score) for the whole study population and broken down by sex, age cohort, marital status, education, ADL and number of chronical diseases.

Visual inspection of the distributions of the LSI-A scores for the whole study population as for stratified subgroups revealed a slightly negative skewness which were confirmed by the Kolmogorov–Smirnov tests for normality.^34^ Therefore, and in addition to the reported quartile scores, deciles for LSI-A score stratified for sex and age decade are presented (see Appendix B).

An attrition analysis was carried out to compare the final study population with the internal attrition. Pearson Chi-squared test was used to analyze statistical differences between these groups. A p-value <0.05 defined statistical significance; all tests were two-sided. Analyses were performed using the SPSS software version 21 (IBM Corporation, Armonk, NY, USA).

## Results

The study population consisted of 2656 (45.7%) men, mean age 69.0 (sd=9.8) years and 3159 (54.3%) women, mean age 70.6 (sd=10.5) years. A little more than a third (36.9%) lived alone and about a quarter (23.3%) had at least one year of university studies. One third (32.9%) were dependent in at least some ADL and about one out of ten (11.6%) suffered from three or more chronical diseases. LSI-A score for the whole study population was 27.8 (sd=6.9). Men scored higher than women; overall 28.5 (sd=6.6) compared to 27.3 (sd=7.1) (Table 1) and also in all decades of age from 60 years to >93 years (Tables 2 and 3). General results for both men and women are that lower scores on LSI-A were associated with increasing age, living alone, a more extensive dependency in ADL and increasing number of chronical diseases. Even though confidence intervals overlap comparing age groups, there is still a clear trend in higher age correlating with lower LS score (Tables 2 and 3).

**Table 2:** LSI-A Norm Values for Men in a General Swedish population in Nine Age Cohorts Between 60–93 Years, n=2656

	n (%)	mn	sd	CI 95%	q1	md	q3	Min–Max	At Ceiling n (%)	L Missing n (%)
Age, (years)										
60	1216 (45.8)	29.4	6.8	29.0–29.8	26.0	31.0	34.0	5–40	15 (1.2)	
66	425 (16.0)	29.1	5.6	28.6–29.7	25.0	30.0	34.0	14–40	2 (0.5)	
72	178 (6.7)	28.4	6.0	27.6–29.3	26.0	30.0	33.0	3–38	-	
78	159 (6.0)	27.5	6.0	26.5–28.4	24.0	28.0	33.0	6–39	-	
81	396 (14.9)	27.6	6.5	26.9–28.2	24.0	29.0	32.5	6–40	3 (0.7)	
84	127 (4.7)	25.6	6.5	24.5–26.8	21.0	26.0	30.0	6–40	1 (0.7)	
87	79 (3.0)	25.8	7.0	24.2–27.4	21.0	27.0	31.0	7–40	2 (2.3)	
90	54 (2.0)	24.5	6.1	22.8–26.1	20.0	24.0	29.0	9–37	-	
93+	22 (0.8)	23.4	7.0	20.3–26.4	17.7	24.5	28.2	11–37	-	
Marital status										12 (0.4)
Living alone	642 (24.2)	25.7	6.8	25.2–26.2	21.0	26.0	31.0	3–40	5 (0.7)	
Cohabiting	2002 (75.4)	29.4	6.2	29.1–29.6	26.0	31.0	34.0	6–40	18 (0.9)	
Education										17 (0.6)
Elementary school	1119 (42.1)	27.7	6.4	27.3–28.0	24.0	29.0	32.0	5–40	6 (0.5)	
Secondary school	834 (31.4)	28.4	6.8	28.0–28.9	25.0	30.0	34.0	3–40	7 (0.8)	
University	686 (25.8)	29.8	6.5	29.3–30.3	27.0	31.0	35.0	5–40	10 (1.4)	
ADL										59 (2.2)
Independent	1893 (71.2)	29.3	6.2	29.0–29.6	26.0	30.0	34.0	3–40	22 (1.1)	
IADL- dependent	408 (15.4)	26.6	7.2	25.9–27.3	21.0	28.0	32.0	5–38	-	
PADL- dependent	296 (11.1)	25.4	7.0	24.6–26.2	20.0	26.0	31.0	7–40	1 (0.3)	
Chronic disease										
None	1007 (37.9)	29.5	6.2	29.1–29.8	21.0	31.0	34.0	3–40	8 (0.8)	
One	957 (36.0)	28.7	6.4	28.3–29.1	25.0	30.0	34.0	6–40	9 (0.9)	
Two	466 (17.5)	27.1	7.2	26.5–27.8	22.0	28.0	33.0	6–40	4 (1.0)	
Three or more	226 (8.5)	25.6	6.7	24.7–26.4	20.0	27.0	31.0	5–40	1 (0.4)

**Table 3:** LSI-A Norm Values for Women in a General Swedish Population in Nine Age Cohorts Between 60–93 Years, n=3159

	n (%)	mn	sd	CI 95%	q1	md	q3	Min–Max	At Ceiling n (%)	Missing n (%)
Age, (years)										
60	1265 (40.0)	29.0	6.8	28.6–29.4	25.0	30.0	34.0	3–40	16 (1.2)	
66	483(15.3)	28.3	6.5	27.8–28.9	25.0	29.0	33.0	4–40	3 (0.6)	
72	236(7.4)	27.0	7.2	26.1–28.0	23.0	28.0	32.0	3–40	2 (0.8)	
78	187(5.9)	25.8	6.7	24.8–26.7	21.0	26.0	31.0	9–40	1 (0.5)	
81	483 (2.6)	25.9	6.8	25.3–26.6	21.0	27.0	31.0	6–40	2 (0.4)	
84	208 (6.6)	24.6	7.1	23.7–25.6	20.0	25.0	30.0	6–40	1 (0.4)	
87	140 (4.4)	22.6	7.2	21.4–23.8	18.0	24.0	28.0	3–38	-	
90	107 (3.4)	24.1	7.4	22.6–25.5	19.0	24.0	30.0	4–40	1 (0.8)	
93+	50 (1.6)	22.9	7.4	20.7–25.0	18.0	23.0	28.4	6–36	-	
Marital status										7 (0.2)
Living alone	1504 (47.6)	25.4	7.3	25.0–25.8	22.0	26.0	31.0	3–40	6 (0.4)	
Cohabiting	1648 (52.2)	28.9	6.5	28.6–29.2	25.0	30.0	34.0	3–40	20 (1.2)	
Education										23 (0.7)
Elementary school	1603 (50.7)	26.0	7.1	25.7–26.4	21.2	27.0	36.0	3–40	10 (0.6)	
Secondary school	862 (27.3)	28.0	7.9	27.5–28.4	24.0	29.0	33.0	3–40	9 (1.0)	
University	671 (21.2)	29.3	6.7	28.8–29.8	25.3	31.0	34.0	6–40	6 (0.9)	
ADL										39 (1.2)
Independent	2008 (63.6)	28.7	6.6	28.4–29.0	25.0	30.0	34.0	3–40	21 (1.0)	
IADL- dependent	298 (9.4)	24.8	7.1	24.0–25.6	20.0	25.0	30.0	8–40	2 (0.6)	
PADL- dependent	814 (25.8)	24.6	7.4	24.1–25.1	20.0	25.0	30.0	3–40	3 (0.3)	
Chronic disease										
None	943 (30.3)	29.5	6.2	29.1–29.9	26.0	30.5	34.0	3–40	16 (1.6)	
One	1058 (34.0)	27.7	6.8	27.3–28.1	23.0	29.0	33.0	3–40	6 (0.5)	
Two	708 (22.7)	25.7	7.2	25.2–26.2	21.0	26.0	31.0	6–40	2 (0.3)	
Three or more	405 (13.0)	24.0	7.8	23.3–24.7	18.0	25.0	30.0	3–40	(0.4)	

Tables 4 and 5 presents LSI-A scores for men and women broken down by age decade and in relation to number of chronical diseases or dependency in ADL. For both men and women and in each decade, lower results on LSI-A was associated with both number of chronical diseases and ADL-dependency. The largest difference in LSI-A scores, between being healthy and having three or more chronic diseases, are for both sexes found among the 60-year olds. A pattern that repeats itself in terms of dependency in ADL although differences in LSI-A scores are small comparing IADL and PADL groups. A clearer distinction is rather between being independent or dependent at all.

**Table 4:** LSI-A norms for Swedish Men by Number of Chronic Diseases and Functional Impairment (Dependent in ADLs) in the decades of 60, 70 and 80 years

Age, Decade	None	Number of Chronic Diseases		Three or More	Independent	ADL	
		One	Two			Dependent in IADL	Dependent in PADL
**60**	n=743	n=601	n=223	n=74	n=1375	n=152	n=88
mn (sd)	30.1 (6.1)	29.4 (6.4)	27.6 (7.2)	25.9 (7.6)	29.7 (6.2)	27.9 (7.3)	25.8 (7.5)
CI 95%	29.6–30.5	28.9–29.9	26.9–28.5	24.2–27.8	29.3–30.0	26.7–29.1	24.2–27.4
q1 md q3	27.0 31.0 34.0	26.0 30.0 34.0	23.0 29.0 33.0	20.0 27.5 32.0	26.0 32.0 34.0	24.0 29.0 34.0	20.0 27.0 32.0
min-max	5–40	8–40	7–40	5–40	5–40	5–38	8–37
at ceiling n (%)	7 (0.9)	8 (1.3)	1 (0.4)	1 (1.3)	17 (1.2)	-	-
**70**	n=108	n=116	n=71	n=42	n=212	n=64	n=45
mn (sd)	28.7 (5.9)	28.6 (5.3)	27.3 (6.6)	25.6 (8.3)	28.4 (5.7)	26.3 (7.4)	27.8 (5.4)
CI 95%	27.6–29.9	27.6–29.6	25.7–28.8	23.6–27.6	27.6 29.2	24.5–28.2	26.1–29.4
q1 md q3	25.6 30.0 33.0	26.0 29.5 33.0	24.0 29.0 31.5	22.0 26.0 30.0	25.0 29.5 33.0	21.5 27.0 32.0	25.0 28.0 32.0
min-max	3–38	13–38	6 −39	6–35	3–39	6–38	15–38
at ceiling n (%)	-	-	-	-	-	-	-
**80+**	n=156	n=240	n=172	n=110	n=306	n=192	n=163
mn (sd)	27.3 (6.2)	26.9 (6.4)	26.5 (7.4)	25.3 (6.3)	28.4 (5.9)	25.6 (7.0)	24.6 (6.9)
CI 95%	26.3–28.2	26.1–27.7	25.7–27.6	24.1–26.5	27.7–29.0	24.6–26-6	23.5–25.7
q1 md q3	23.0 28.0 32.0	22.0 28.0 32.0	21.0, 28.0, 32.0	21.0 26.5 30.0	25.0 29.0 32.0	20.5 27.0 31.0	19.0 25.0 30.0
min-max	11–40	6–40	7–40	11–38	9–40	6–38	7–40
at ceiling n (%)	1 (0.6)	1 (0.4)	4 (2.2)	-	5 (1.6)	-	(0.6)

**Table 5:** LSI-A norms for Swedish Women by Number of Chronic Diseases and Functional Impairment (Dependent in ADLs) in the Decades of 60, 70 and 80 years

Age, Decade	None	Number of Chronic Diseases		Three or More	Independent	ADL	
		One	Two			Dependent in IADL	Dependent in PADL
**60**	n=641	n=630	n=323	n=154	n=1334	n=85	n=310
mn (sd)	30.7 (5.6)	28.7 (6.4)	26.6 (7.6)	25.6 (7.6)	29.6 (6.4)	26.6 (7.4)	26.2 (7.1)
CI 95%	30.3–31.2	28.2–29.2	25.8–27.5	24.4–26.9	29.3–30.0	25.0–28.2	23.4–27.0
q1, md, q3	28.0 32.0 35.0	25.0 30.0 34.0	22.0 27.0 32.0	21.0 27.0 32.0	27.0 32.0 34.0	22.0 27.0 32.5	23.0 27.0 32.0
Min-max	3–40	4–40	6–40	4–40	6–40	8–40	3–39
At ceiling n (%)	14 (2.1)	2 (0.3)	2 (0.6)	1 (0.6)	17 (1.3)	2 (2.3)	-
**70**	n=106	n=141	n=96	n=80	n=273	n=36	n=106
mn (sd)	27.7 (5.7)	27.3 (7.0)	26.3 (7.1)	23.7 (7.8)	27.4 (6.8)	25.0 (7.2)	24.2 (7.0)
CI 95%	26.6–28.8	26.2–28.4	24.9–27.7	21.9–25.4	26.3–28.2	22.6–27.5	22.8–25.5
q1 md q3	23.0 29.0 31.0	22.0 28.0 32.0	21.0 26.3 32.0	17.2 26.0 30.0	22.5 29.0 32.0	21.2 25.5 30.8	18.8 25.1 30.0
Min-max	9–40	8–40	9–38	3 −38	3–40	9–37	8–38
At ceiling n (%)	1 (0.9)	2 (1.4)	-	-	3 (1.1)	-	
**80+**	n=196	n=287	n=289	n=216	n=401	n=177	n=398
mn (sd)	26.4 (6.9)	25.6 (6.9)	24.4 (6.7)	23.0	26.6 (6.5)	23.8 (6.8)	23.5 (7.4)
CI 95%	25.5–27.4	24.8–26.4	23.7–25.2	21.9–24.0	26.0–27.2	22.8–24.8	22.8–24.3
q1, md, q3	23.0 27.0 32.0	21.0 26.0 31.0	19.0 25.0 29.0	17.0 23.0 29.0	23.0 27.0 32.0	19.0 24.0 32.2	18.0 24.0 29.0
Min-max	6–40	3–40	6–39	3 −40	6–40	9–37	3–40
at ceiling n (%)	1 (0.5)	2 (0.6)	-	1 (0.4)	1 (1.2)	-	3 (0.7)

Healthy participants, including both men and women, who are independent in ADLs (n=1545) scored 30.1 (sd=5.8) on LSI-A. Those suffering from at least one chronic disease but independent in ADLs (n=2365) scored 28.3 (sd=6.6). Healthy participants with an ADL-dependency (n=376) scored 27.1 (sd= 6.8), while those suffering from at least one chronic disease and dependent in I- or PADL (n=1440) scored 24.7 (sd= 7.3)

There were no floor effects but several, although weak, ceiling effects ie, proportion of subjects receiving the maximum score of 40 points. The largest proportion, 2.2%, was found among men 80+ years of age and suffering from two chronical diseases.

Results from analysis of the internal attrition revealed significantly larger proportions of the oldest cohorts, women, those living alone and those with lower education in the attrition group, compared to study population (data not shown).

## Discussion

As already suggested, the major clinical utility of the current study is that it provides norm values of LSI-A that could be a guide in comparing LS for men and women 60 years and older from the general population as well as for groups suffering from illnesses or functional impairment. The strengths of this study are the large sample size, the stratification for age and sex and that the population represents both urban and rural areas, thereby increasing generalization. A stratification of the study population in further socio-demographic variables, social relations, personal finances, or specific medical, psychological or functional conditions, ie, much of what has previously been shown to be associated with LS, was not within the scope of this work. However, to briefly comment on this study, we can see that the results are largely in line with what has been reported previously. Men scored higher than women; lower age, living in a relationship, higher education, being healthy and independent in ADLs were all associated with a better LS.^9^,^25^,^35–39^ A pattern that also appears is that functional dependence seems to affect LS more than morbidity per se. Looking at the whole study population we found that participants suffering from one or more diseases but independent in ADLs scored 28.3 points, which is somewhat higher than those who were healthy but dependent in ADLs scoring 27.1 points. The constraints that dependency in ADL causes probably affects LS more than a chronic disease which one learns to cope with and which does not involves any ADL dependency.

An absolute requirement for the data to be regarded as normative is that they really represent the population they intend to reflect. In this study, participants were randomized from the National Population Register and we have no reason to believe that any sampling bias would exist even though some restrictions had to be considered in the composition of the final study population. As mentioned above, to ensure the reliability of the answers given in the LSI-A questionnaire, participants diagnosed with dementia or assessed as severely cognitively impaired, the latter based on the results from MMSE, were excluded. One might argue that this was to be too restrictive. In a previous study from the GÅS-project exploring associations between symptoms and LS among men and women aged 78–93 years where demented participants were included, no significant difference in LSI-A scores between dementia and non-dementia were shown.^10^ Furthermore, cognitive function like processing speed and spatial ability, expression of fluid intelligence, predict LS in a 3-year follow-up, but the attributable fraction to LS is small, less than 3%.^19^

The attrition analysis revealed that subjects age 80+, women, single-living and those with only elementary school were somewhat, but significantly, overrepresented in the dropout group compared to respectively 60 and 70 year olds, men, those co-habiting and those with secondary school or university education. That is, participants in groups already scoring low on LSI-A tended to a greater extent not to participate and the largest attrition was seen among the oldest (80+), something that is not uncommon in studies aimed at the elderly.^40^ However, we believe that it is highly unlikely that results would have been much different in these groups as regards to the final study sample.

A shortcoming of cross-sectional studies like this, except that we cannot say anything about influences from the past, future hypothesis or causality in general, is the risk of cohort effects. The trend that life satisfaction deteriorates with increasing age, number of chronical or functional impairment seems stable, but we cannot exclude that in addition to age or illness, environmental, socio-demographics or other circumstances may have affected people differently during certain periods of time,^41^ which might have influenced participants in assessing LS. Furthermore, the study was conducted in Sweden and generalization of data to other countries may not be applicable to other countries.

Moreover, the perception of what is meant by LS in general and what is considered important in the assessment of LS changes during lifetime. Individuals can use different scales or benchmarks based on their situation in general, changes in health status or time in life when assessing LS.^6^ It is therefore important to be aware that the measurements of LS are a relative concept and that comparisons between gender, age cohorts or other groups should be done with the utmost caution.

## Conclusions

Norm values here presented may facilitate assessments and evaluation of life satisfaction in the general elder population and as reference values to clinical trials. Female sex, rising age, morbidity and impaired functional ability were all associated with impaired LS.
